# The novel BTB-kelch protein, KBTBD8, is located in the Golgi apparatus and translocates to the spindle apparatus during mitosis

**DOI:** 10.1186/1747-1028-8-3

**Published:** 2013-04-11

**Authors:** Sandra Lührig, Susanne Kolb, Nadine Mellies, Jessica Nolte

**Affiliations:** 1 Institute of Human Genetics, University of Göttingen, Göttingen, 37073, Germany

**Keywords:** Kbtbd8, BTB-kelch, Golgi, Spindle apparatus

## Abstract

Proteins of the BTB-kelch family are known to be involved in multiple biological processes such as migration, cytoskeleton arrangement, regulation of cell morphology, protein ubiquitination and gene expression. KBTBD8 is a new member of this family. The gene was found in a comparative transcriptome analysis of pluripotent stem cells and was therefore suggested to play a role in the regulation of pluripotency. Comparative analysis of the gene and protein sequences revealed a high conservation throughout evolution especially in the characteristic domains of BTB, BACK and kelch. We identified the Golgi apparatus as the subcellular localization of the KBTBD8 protein in non-dividing cells and could show that KBTBD8 co-localizes with α-tubulin on the spindle apparatus of mitotic cells suggesting a role in cell proliferation. In conclusion, KBTBD8 is a new member of the BTB-kelch superfamily that is located in the Golgi apparatus and translocates to the spindle apparatus during mitosis.

## Background

**Figure 1:**
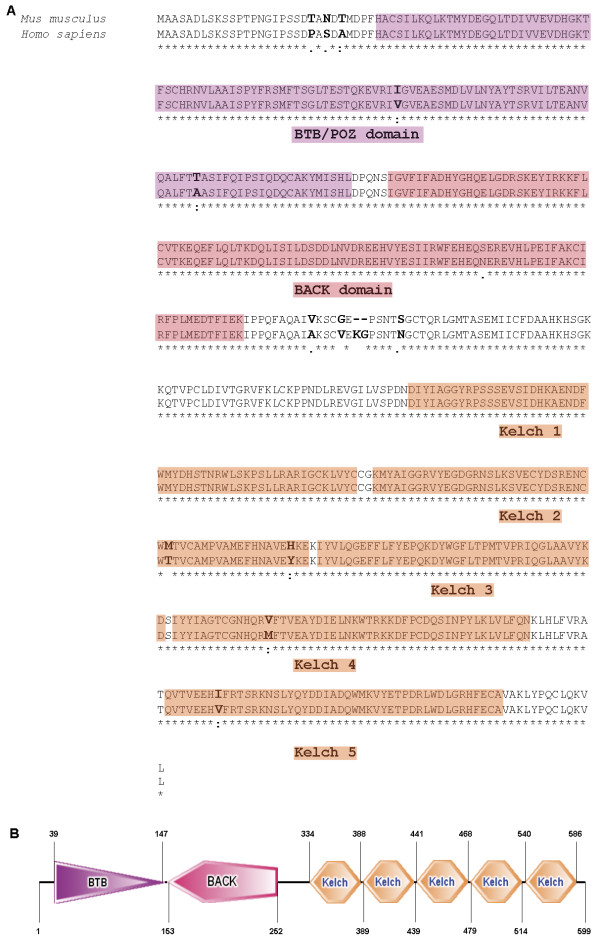
**Structure of KBTBD8 protein. A**: Comparison of the amino acid sequences of mouse and human KBTBD8 protein including domain structures. Amino acid sequence of mouse and human KBTBD8 protein shows a 95% homology. Both consist of five kelch motifs (orange) at the N-terminal part of the protein. The C-terminal part has a BTB/POZ-domain (purple) followed by a BACK-domain (red). Asterisk (*) indicates positions which have a single, fully conserved residue. Colon (:) indicates conservation between groups of strongly similar properties. Period (.) indicates conservation between groups of weakly similar properties. **B**: Domain structure of mouse KBTBD8 protein including the depiction of amino acid position.

In a comparative study of the transcriptomes of mouse pluripotent Embryonic Stem Cells (ESCs) and multipotent adult Germline Stem Cells (maGSCs) [1], Kbtbd8 (kelch repeat and BTB (POZ) domain containing 8) was found to be highly expressed in undifferentiated and differentiated stem cells. Therefore we suggested that the unknown gene Kbtbd8 might play a role in pluripotency. The analysis of the gene and protein structure revealed KBTBD8 as a member of the BTB-kelch family of proteins. It is highly conserved through evolution and the protein consists of a BTB/POZ domain, a BACK domain and five kelch repeats (Figure 1).

The BTB/POZ domain was first identified in *Drosophila melanogaster*. It displays a conserved motif found in many bric-á-brac, tramtrack and complex transcriptional regulators as well as in several pox virus zink finger proteins [2-4]. There are five subfamilies of kelch-repeat family known and Kbtbd8 can be classified as a member of the “N-dimer, C-propeller” subfamily [5]. These proteins contain the BTB/POZ domain at the N-terminus and four to six kelch motifs at the C-terminus. Furthermore KBTBD8, as most of the BTB-kelch proteins, also contains a carboxyterminal kelch, a BACK domain (Figure 1). Proteins belonging to this subtype are also known as BBK (BTB, BACK, kelch) proteins [6]. The BTB domain in these proteins has been shown to be responsible for protein dimerisation and oligomerization in accordance to the often found function of actin filament organisation [7-10]. The ENC-1 protein was shown to be an actin-binding protein that is involved in the organization of the actin cytoskeleton [11], gigaxonin was shown to serve as a functional link between the intermediate filaments of the cytoskeleton and actin filaments [12]. Despite the connection to the actin cytoskeleton, several other functions of members of the BTB-kelch family are known. Keap1 for instance was shown to be a transcriptional regulator by directly binding the transcription factor Nrf2 via its Kelch-repeats [13]. The function of BTB-kelch proteins in regulation of transcription was further supported by the finding that two BTB proteins directly interact with topoisomerase I, even though a direct regulation of the enzyme activity could not be demonstrated [14]. Geyer et al. [15] identified BTB/POZ proteins in yeast as components of ubiquitin ligase complexes. This was supported by the results of other approaches in C. elegans [16-18]. Taken together, BTB-kelch proteins play an important role in multiple biological processes, are highly conserved and therefore seem to be important even in evolution.

To date there is only one member of this family known that is located in the Golgi apparatus. The BTB-kelch protein “leucin zipper-like transcriptional regulator 1” (LZTR-1) was first found to be hemizygously deleted in DiGeorge syndrome patients [19]. In 2006, Nacak et al. [20] characterized LZTR-1 on the molecular level and found the protein to be Golgi-matrix associated. Further analysis revealed that the BTB domain is responsible for this association.

Here we have characterized another BBK family member and show that KBTBD8 is specifically localized at the Golgi in non dividing cells and becomes restricted to the spindle apparatus upon mitosis. To our knowledge this is the first time that a BBK family member is shown to be trapped in the Golgi and becomes translocated to the forming spindle apparatus upon mitosis.

## Results

### Expression analysis of mouse and human KBTBD8

Mouse Kbtbd8 is located on chromosome 6 and is predicted to have two protein coding transcripts. Transcript one consists of 4 exons, transcript 2 is lacking exons 1 and 2 (Additional file 1: Figure S1). This leads to a truncated BTB/POZ domain of the isoform b. The open reading frame (ORF) of the full length isoform is 1801 bp in size and encodes a 599 AA protein with a predicted size of 68.6 kDa. The ORF of the shorter transcript is 1569 bp in size and encodes a 522 AA protein with a predicted size of 60.4 kDa. Human KBTBD8 is located on the short arm of chromosome 3, consists of 4 exons and the ORF of 4684 bp encodes for a 601 AA protein with a predicted size of 68.8 kDa.

**Figure 2:**
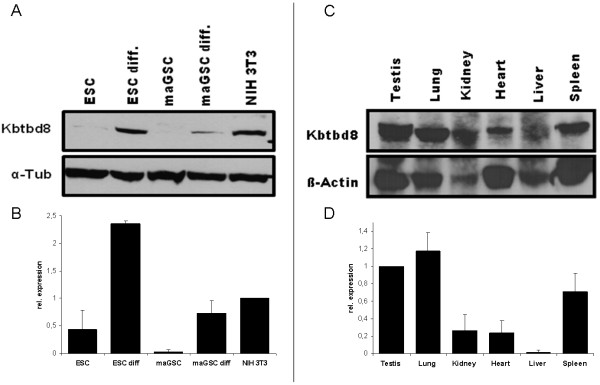
**Quantitative expression of KBTBD8 on mouse tissues and cell lines. A**: Western Blot analysis on mouse cell lines using a Kbtbd8 specific antibody revealed a high protein expression of KBTBD8 in differentiated ESCs and maGSCs as well as in NIH3T3 cells and a low one in ESCs. However, in maGSCs no KBTBD8 protein could be detected. α-Tubulin served as loading control. **B**: qRT-PCR results support the Western Blot results. **C**: Western Blot analysis on different mouse organs revealed an ubiquitous expression in all tested organs. β-Actin served as loading control. **D**: qRT-PCR results support the Western Blot results. Abbreviations: ESC: Embryonic Stem Cell; ESCdiff.: differentiated Embryonic Stem Cells; maGSC: multipotent adult Germline Stem Cell; maGSCdiff.: differentiated multipotent adult Germline Stem Cell.

We initiated the expression analysis of mouse Kbtbd8 by RT-PCR using primers that can detect both predicted transcripts and since we expected Kbtbd8 to be specifically expressed in pluripotent stem cells, we started with the analysis of organs and different pluripotent stem cells. Indeed, we could find the expected two transcripts in tissues (testis, muscle) and cell lines (ESC, maGSC, iPSC, F9) (Additional file 1: Figure S1). We further analyzed the level of Kbtbd8 expression by qRT-PCR (Figure 2B + D) and because an antibody was available (Abcam, ab103727) we proceeded the expression studies by Western Blot analysis (Figure 2A + C). Kbtbd8 mRNA expression could be detected in all tested cell lines and organs. However, KBTBD8 protein could be only detected in cells and organs which showed a high mRNA expression level. The second predicted isoform of the KBTBD8 protein could not be detected. Nevertheless, expression analysis on RNA as well as on protein level in mouse tissues and cell lines revealed an ubiquitous expression of KBTBD8. This result could be supported by RT-PCR on selected human tissues and cell lines (Additional file 2: Figure S2A) where the predicted transcript could be found in most of the tested samples.

**Figure 3:**
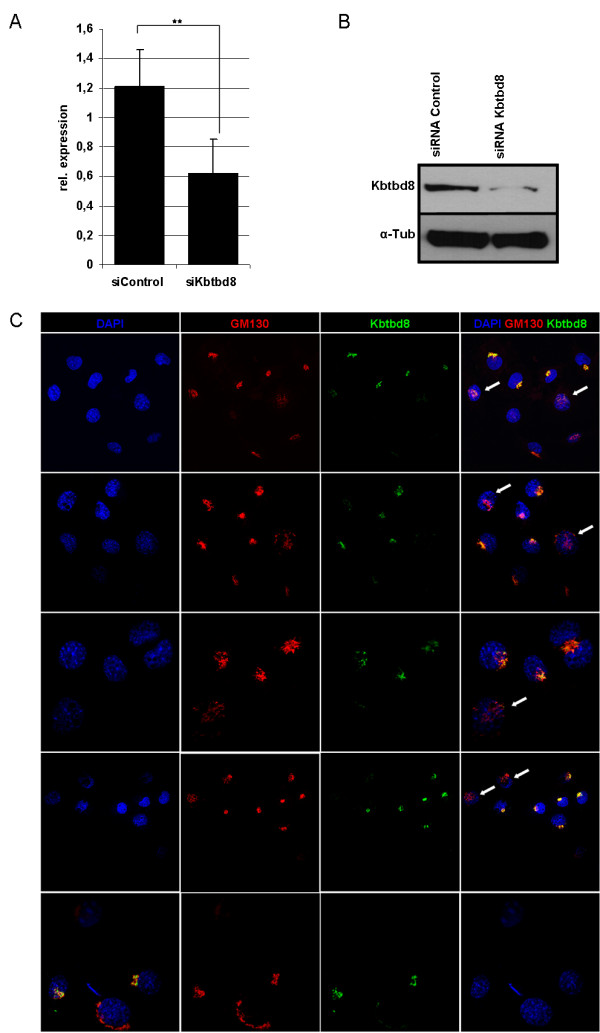
**Altered KBTBD8 expression results in the dislocation of Golgi structures.** To test the specificity of the KBTBD8 antibody and to see, if there is an effect on the Golgi apparatus, NIH3T3 cells where transfected with siRNA against Kbtbd8. **A**: qRT-PCR results followed by statistical analysis using *t*-test revealed the significant downregulation of Kbtbd8 mRNA in two biological replicates. **B**: Western Blot results support the downregulation of KBTBD8 even on the protein level. **C**: Immunocytochemistry using GM130 and KBTBD8 specific antibodies revealed an altered localisation of the Golgi matrix protein GM130. In cells, were KBTBD8 expression is downregulated or absent upon siRNA transfections, the localization of the GM130 signal becomes rearranged (white arrows).

To test the specificity of the antibody before starting deeper analysis, we performed siRNA mediated downregulation of Kbtbd8 in NIH3T3 cells. Analysis of the mRNA level revealed a downregulation up to 60% resulting in a clear reduction of the protein as shown by Western Blot analysis (Figure 3A + B). Immunocytochemical analysis of siRNA transfected cells revealed a shift of the Golgi apparatus in the cells, where KBTBD8 was clearly downregulated or absent (Figure 3C).

### Cellular localization of mouse and human KBTBD8

**Figure 4:**
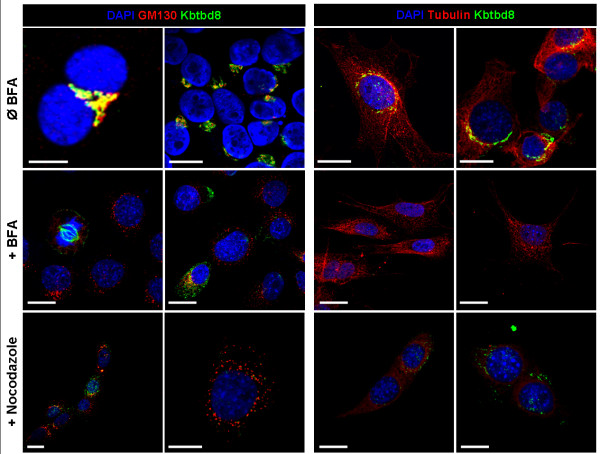
**Localization of KBTBD8 protein in NIH3T3 cell and after treatment with BFA or Nocodazole.** NIH3T3 cells – with and without BFA or Nocodazole treatment - were fixed and co-stained with KBTBD8 antibody and Golgi matrix marker GM130 or microtubule marker protein α-Tubulin. Treatment with Brefeldin A (BFA) resulted in resorption of the Golgi membrane and Golgi marker proteins to the ER. For GM130 the expected punctuated cytoplasmic localization could be found indicating that the treatment worked. However, KBTBD8 was either no longer detectable, not co-localizing with GM130 or associated to the spindle apparatus of mitotic cells. Co-staining with α-Tubulin and Kbtbd8 in untreated cells showed no localisation of KBTBD8 at the cytoskeleton. After Nocodazole- treatment, which results in the depolarisation of microtubules and in the disperse of the Golgi stacks to the cytoplasm, GM130 and α-Tubulin showed the expected localisation whereas KBTBD8 was again either not detectable at all or no longer co-localizing with GM130. These results indicate that KBTBD8 is not a part of the Golgi matrix. Scale bar, 10 μm.

**Figure 5:**
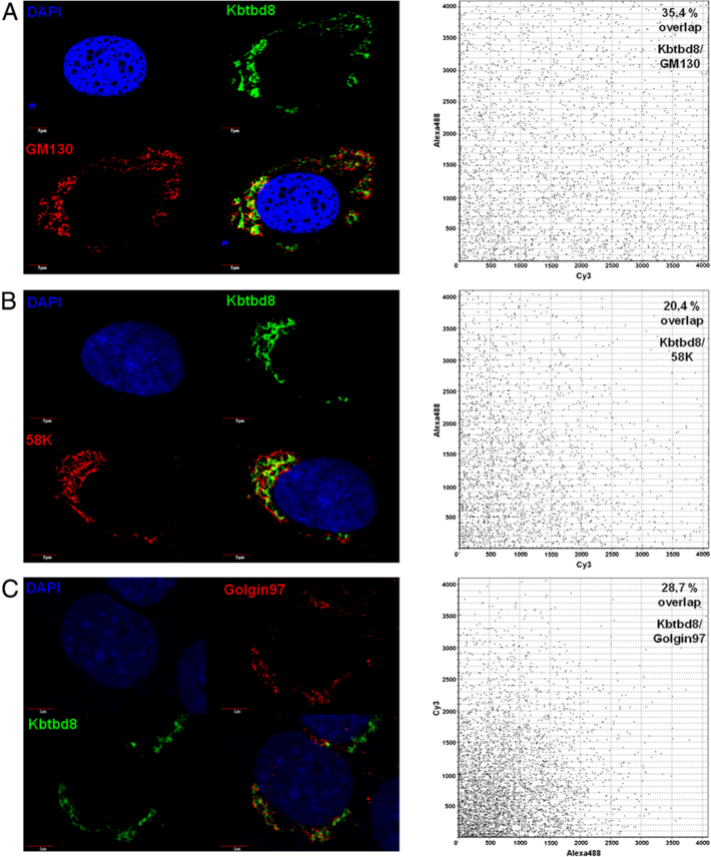
**Co-localization of KBTBD8 and GM130, 58 K and Golgin97.** BJ cells were fixed and co-stained with KBTBD8 antibody and the Golgi markers GM130 (**A**), 58 K (**B**) or Golgin97 (**C**). Overlapping of the two fluorescence pictures was analyzed using the Fluoview Software driving the CONFOCAL LASER SCANNING BIOLOGICAL MICROSCOPE (Olympus). **A**: Co-staining of KBTBD8 and GM130 showed a 34.4% co-localisation. **B**: Co-staining of KBTBD8 and 58 K showed a 20.4% co-localisation. **C**: Co-staining of KBTBD8 and Golgin97 showed a 28.7% co-localisation. Scale bars as indicated.

**Figure 6:**
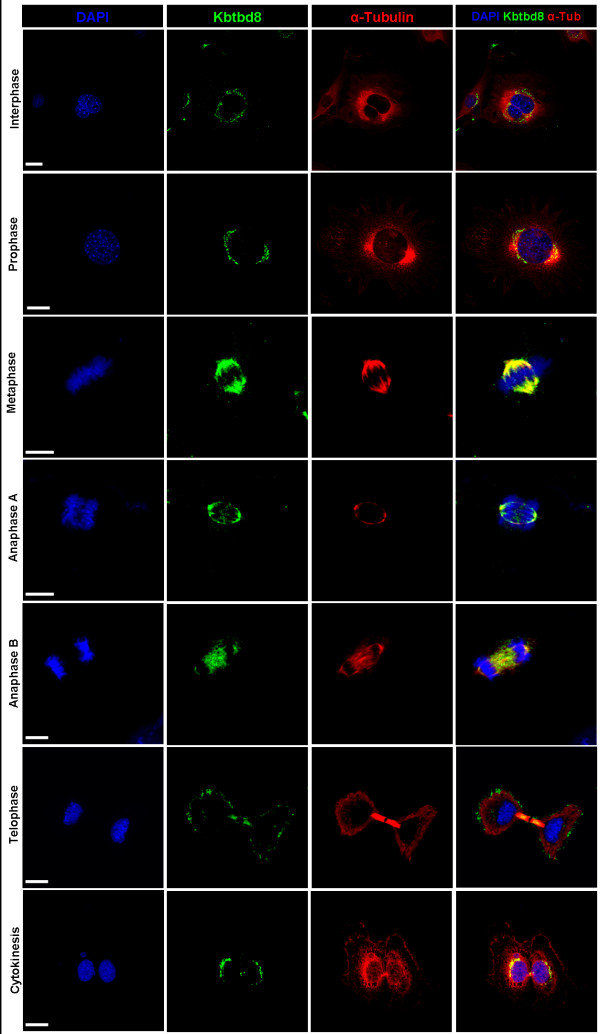
**KBTBD8 is associated with the spindle apparatus in dividing cells.** Proliferating NIH3T3 cells were fixed and co-stained with anti-KBTBD8 and anti- α-Tubulin antibodies. KBTBD8 seems to be released from the Golgi stackles with the onset of mitosis and co-localizes with α-Tubulin even though the fibres of the spindle apparatus are not so obvious.

To elucidate the cellular compartment in which KBTBD8 is localized, we performed immunocytochemical analysis. The staining showed a typical perinuclear staining which encouraged us to hypothesize that KBTBD8 is either localized in the endoplasmic reticulum (ER) or in the Golgi apparatus. We started with GM130 staining, as this protein is a well known marker for the Golgi complex [21], and indeed could show, that KBTBD8 colocalize with GM130 in NIH3T3 cells (Figure 4, upper panel). This result could be confirmed in human fibroblasts as well (Figure 5A). To further confirm the Golgi apparatus as the compartment in which KBTBD8 is localized, we used two other Golgi specific markers (58 K and Golgin97) for co-staining (Figure 5B + C) and again could identify the Golgi as the subcellular localization of our protein. As it was obvious that the percentage of overlapping staining was not 100% we quantified the areas of co-staining by LSM software and found a co-staining for KBTBD8 and GM130 in 35.4%, of KBTBD8 and 58 K in 20.4% and of KBTBD8 and Golgin97 in 28,7% of the Golgi stackles. This result leads us to hypothesize that KBTBD8 is not associated to the Golgi membranes as it is known for GM130, 58 K and Golgin97. To clarify the localization within the Golgi complex, we next treated NIH3T3 cells with Brefeldin A (BFA) and Nocodazole. BFA is a fungal antibiotic that reversibly blocks the protein transport from the ER to the Golgi [22] and therefore causes a collapse of the Golgi stacks. In consequence of BFA treatment, the Golgi membranes and marker enzymes are redistributed back to the ER, coat proteins to the cytoplasm and Golgi matrix associated proteins to Golgi debris as well as ER exit sites [23]. Therefore, BFA treatment of cells results in a punctuated arrangement of Golgi fragments that can be shown by GM130 staining. Nocodazole promotes the depolymerisation of microtubuli. As the perinuclear localization of the Golgi apparatus is due to dynamic but not stable microtubules, the treatment with Nocodazole leads to a disruption of the Golgi structure and therefore to the distribution of Golgi ministacks throughout the cytoplasm [24]. Treatment of NIH3T3 cells with both, BFA and Nocodazole, resulted in the expected punctuated dispersion of GM130 (Figure 4), but KBTBD8 relocation upon treatment did not follow a typical pattern for Golgi-resident proteins. In both, BFA and Nocodazole treated fibroblasts, KBTBD8 was either distributed all over the cells without preferred colocalization with GM130 or not detectable at all or localized at the spindle apparatus of mitotic cells. This results suggest that KBTBD8 is not a Golgi matrix protein and shows no direct interaction to microtubules. Furthermore a co-staining of KBTBD8 and α-tubulin showed no co-localization in non-dividing cells suggesting the specificity of co-localisation for dividing cells. However, to clarify whether the specific staining pattern at the spindle apparatus of mitotic cells seen in BFA treated cells is due to the treatment, we repeated the experiment with serum starvation synchronized NIH3T3 cells and indeed could find the specific staining at the spindle of dividing cells in all mitotic stages (Figure 6) revealing that the spindle associated localization is indeed specific for dividing cells.

## Discussion

Proteins of the BTB-kelch family are known to be involved in multiple biological processes such as migration, cytoskeleton arrangement, regulation of cell morphology, protein ubiquitination and gene expression [5]. Therefore a prediction about the function of a new member of this family is difficult. Here we describe a novel BTB-kelch protein, KBTBD8 that is highly conserved from mouse to human. We report that 1) Kbtbd8 is ubiquitiously expressed; 2) KBTBD8 is a novel protein of the BTB-kelch family that is located in the Golgi apparatus; 3) KBTBD8 is not a Golgi-resident protein and 4) KBTBD8 is the first BTB-kelch protein that localizes on the spindle apparatus upon mitosis in proliferating cells.

There are many BTB-subfamilies known that are distributed through species but the quantity of families and their members varies between species (reviewed in [6]). Whereas for *Saccharomyces cerevisiae* there are only four subfamilies with six proteins known, *Drosophila melanogaster* already harbours eight subfamilies with 85 proteins. *Mus musculus* and *Homo sapiens* have the same eight subfamilies with 194 and 183 total proteins, respectively. The subfamilies are known as BTB only, T1-Kv, ElonginC, Skp1, MATH-BTB, other architectures, BTB-ZF and BBK whereas the last two resemble the largest groups with more than 40 proteins in mouse and human.

BTB-ZF (BTB-Zinc finger) proteins are also known as POK (POZ and Krüppel zinc finger) proteins [6]. As expected for zinc finger proteins, many members of this family were characterized as transcription factors. The BTB- domain in these proteins is responsible for dimerisation or even for heteromeric BTB-BTB associations whereas the ZFs are responsible for DNA binding.

The BBK (or BTB-BACK-kelch) family resembles the second large group within the BTB proteins. The architecture of these proteins, the BTB/POZ domain together with the BACK domain at the N-terminal part of the proteins and the kelch-repeats at the C-terminal part of the proteins, allows on the one hand the dimerization via the BTB domain and on the other hand the association to cytoskeleton via their kelch-repeat domain, which is known for many BBK proteins [5]. As Kbtbd8 belongs to this family and we could observe faint cytoskeleton staining in rare cases, we co-stained with tubulin (Figure 4) in Nocodazole treated and untreated cells but could not confirm this assumption. Therefore, Kbtbd8 seems not to be associated to the cytoskeleton.

In non-dividing cells, the Golgi apparatus is located at the periphery of the nucleus, close to the ER. During cell division the Golgi needs to be segregated and distributed to the two daughter cells. With the onset of mitosis, the Golgi complex become fragmented and disperses throughout the cell. The Golgi matrix associated proteins like GM130 mark this dispersion and can be found as punctuated structures throughout the cytoplasm. Treatment of cells with Brefeldin A (BFA) has the same effect: it blocks the protein transport from the ER to the Golgi [22] and therefore causes a collapse of the Golgi stacks resulting in punctuated structures. Furthermore it was shown that treatment with BFA had no effect on partitioning of Golgi matrix proteins during mitosis. GM130 positive matrix fragment become associated with the forming spindle poles in pro- and metaphase stages. In anaphase cells, these fragments were found to move with the separating sister chromatids and end up on both sides of each new-forming nucleus [25]. Furthermore, it was shown that the spindle plays a direct role in the inheritance of the Golgi [26].

To our knowledge there is only one other BTB-kelch protein known, that shows the golgi compartment as its subcellular localization: “leucin zipper-like transcriptional regulator 1” (LZTR-1). Nacak et al. [20] characterized LZTR-1 and could show a Golgi matrix associated behaviour of LZTR-1 upon BFA treatment and therefore conclude that LZTR-1 is, as GM130, a golgi matrix protein. They further analyzed the domains concerning their function and could show that the BTB domain is responsible for the localization in the golgi apparatus. Since KBTBD8 shows a different behaviour upon BFA treatment than GM130, we conclude that KBTBD8 is not a matrix associated Golgi protein but trapped in the lumen. These results fit to the observation that KBTBD8 does not show a 100% co-localisation with GM130, 58 K and Golgin97. Upon dissociation of the Golgi complex during the entry into mitosis, KBTBD8 seems to be released and translocated to the spindle apparatus as we could show by co-staining with α-tubulin on mitotic cells.

Although there is no protein from the BTB-kelch family known that shows a connection between the Golgi complex and the microtubule network, other proteins have been shown to be associated with both, the cytoskeleton – either actin filaments or microtubules - and the Golgi. These proteins include members of the Hook families [27], cytoplasmic linker proteins (CLIPs) [28], and plakins [29]. The Microtubule actin crosslinking factor 1b (MACF1b) was shown to be a linker protein between the actin filaments and the microtubules. However, MACF1b is - despite its localization to the cytoskeleton – mainly located in the Golgi and it could be shown that the altered expression of MACF1b mediated by RNA interference resulted in the dispersal of the Golgi complex. A similar dislocation of Golgi structures could be also seen by KBTBD8 downregulation. These results lead us to suggest that KBTBD8 plays an important role in the maintenance of the Golgi structure. Whether altered KBTBD8 expression will also affect the mitotic spindle, needs to be elucidated in the future.

## Conclusion and outlook

KBTBD8 is the first described BBK protein that is located in the Golgi apparatus and translocates to the forming spindle upon entry into mitosis. The localization pattern led us to hypothesize that it plays a role in golgi/mitotic functions. Further studies will be performed in the future to elucidate, whether Kbtbd8 has an influence on proliferation/ cell cycle by effecting the configuration of the spindle apparatus. For LZTR-1 it was shown that the BTB/POZ domain is responsible for the Golgi localization [20]. Therefore we suggest that it is also true for Kbtbd8. This assumption has to be investigated and confirmed in future experiments and would mean that there is a new unknown role for the BTB domain besides di- and oligomerisation, namely the localization within the Golgi apparatus.

## Material and methods

### Cell lines, cell culture and BFA treatment

Human skin fibroblast cell line BJ was purchased from Stemgent (Cambridge, USA). NIH3T3 cells and BJ cells were cultured in standard fibroblast medium consisting of DMEM supplemented with 10% FBS and 1% pen/strep.

Brefeldin A was purchased from AppliChem (Darmstadt, Germany). BFA was dissolved in EtOH and used at a final concentration of 1 μg/ml. Incubation with BFA containing medium was carried out for 60 min. Nocodazole was purchased from Sigma-Aldrich (Deisenhofen, Germany). Nocodazole was dissolved in DMSO and used at a final concentration of 10 μM for 2 h. Cells were than directly fixed and used for immunofluorescence staining.

### Immunofluorescence staining

Cells were fixed with 4% paraformaldehyde (PFA) in PBS (pH 7.4) for 10–15 min at room temperature. Fixed cells were permeabilized with 0.1% Triton X-100 for 5 min and than blocked with 1% BSA (Sigma-Aldrich, St. Louis, USA)/PBS-buffer for at least 1 h before being incubated overnight with the appropriate primary antibodies specific for GM130 (BD Laboratories), 58 K (Abcam), Golgin97 (Molecular Probes, kindly provided by Prof. S. Hoyer-Fender, Göttingen), Kbtbd8 (Abcam) or α-Tubulin (Sigma-Aldrich).

Cells were rinsed three times and incubated in the appropriate Cy3-or Alexa488-conjugated secondary antibody (Sigma, Invitrogen, respectively). For negative control, cells were incubated with IgG as first antibody. All incubations were performed in PBS (pH 7.4) with 5% BSA and 0.1% Triton X-100. For nuclear staining, fixed cells were imbedded with DAPI (40, 60-diamidino-2-phenylindole) (SIGMA) dye. Microscopy was performed on an Olympus confocal laser scanning microscope (LSM). Pictures were taken as individual confocal stacks.

### Origin of cells and tissues used in Western Blot and RT-PCR analysis

Mouse organs were taken from animals according to national regulations for the Care and Use of Laboratory Animals (similar to the U.S. National Research Council guidelines). Isolation and origin of ESC and maGSC cell lines was described previously [30]. iPSCs were a kind gift of Rudolf Jaenisch [31]. F9, NIH3T3, Hela and MDA-MB 231 were purchased from ATCC (LGC Standards, Wesel, Germany). MEFs (mouse embryonic fibroblasts) were isolated according to standard mouse ESC culture conditions. RNA from human tissues was purchased from BD Bioscience CLONTECH (Human Total RNA Master Panel II) and used directly for cDNA synthesis.

### Isolation of proteins and Western Blot

Cells or tissues were resuspended in lysis buffer (10 mM Tris/HCl, pH 8, 1 mM EDTA, 2.5% SDS) supplemented with 1 mM phenylmethanesulphonylfluoride (PMSF) and protease inhibitors and than sonicated. Protein extracts (20 μg) were denaturized at 70°C in NuPage SDS sample buffer (Invitrogen), separated on a NuPage 10% Bis-Tris Gel (Invitrogen) and transferred to a Hybond-C Extra membrane (GE Healthcare Europe). Blots were blocked for unspecific binding with 5% nonfat dry milk in blocking buffer (25 mM Tris, 0.15 M NaCl, 0.1% Tween20) and were incubated overnight at 4°C with primary and - after washing in blocking buffer for 1 h at 4°C - with secondary HRP-conjugated antibody. Protein bands were visualised using enhanced chemiluminescence as described by the manufacturer (Santa Cruz Biotechnology).

### RNA isolation and RT-PCR

Total RNA was extracted from cells or tissues by using NucleoSpin® RNA II (Macherey & Nagel) according to manufacturer’s protocol. Prior to reverse transcription using Superscript II cDNA synthesis kit and oligo(dT) primer (Invitrogen), RNA was treated with DNase I (Sigma) to avoid any genomic DNA contamination. RT-PCR amplification was performed by using specific primers for mouse and human Kbtbd8, mouse HPRT and human TBP. RT-PCR was done with 30–33 cycles of 94°C, 30 s; 50°C–62°C, 30 s; 72°C, 45 s, depending on the primer sets. Primersequences: mKbtbd8 (F: 5^′^-CGC AAT GAC ATT TCC TCT TT-3^′^; R: 5^′^-GGA AGG AAT TTG GAA GAT GCT-3^′^); mHPRT (F: 5^′^-CGT CGT GAT TAG CGA TGA TG-3^′^;R: 5^′^-TAT GTC CCC CGT TGA CTG AT-3^′^); hKbtbd8 (F:5^′^-ATG GGA TTC CAT CTT CAG ACC-3^′^; R: 5^′^-TTT CTG TAA GGC CGC TAG TGA-3^′^); hTBP (F: 5^′^-AGC CTG CCA CCT TAC GCT CAG-3^′^; R: 5^′^-TGC TGC CTT TGT TGC TCT TCC-3^′^).

### Quantitative RealTime-PCR (qRT-PCR)

RNA was isolated and treated as described in the former section. Quantitative RealTime-PCR was performed on ABI Prism 7900 HT Fast Detection System (Applied Biosystems Inc.). Each 10 μl reaction was performed in 384-well format using SYBRgreen PCR Master Mix (Qiagen) and 3 μM of each PCR primer. All reactions were performed in triplicate on one plate and repeated three times (technical replicates). For each experiment a second biological replicate was performed to reach statistical significance. Levels of mRNA expression were normalized to those of the mouse housekeeping gene HPRT (Hypoxanthine-guanine phosphoribosyltransferase). Oligonucleotide primers for qRT-PCR were obtained from Eurofins MWG Operon (Ebersberg, Germany). Primer sequences were as follows: qRT-HPRT (F: 5^′^-AGC CCC AAA ATG GTT AAG GTT GC-3^′^; R: 5^′^-TTG CAG ATT CAA CTT GCG CTC AT-3^′^); qRT-Kbtbd8 (F: 5^′^-GCA ATC AGC CCC TAC TTC -3^′^; R: 5^′^-CAC TGG TCT TGT ATG GAA GG-3^′^).

### Transfection of NIH3T3 cells with siRNA

siRNA for mouse Kbtbd8 was purchased from SantaCruz (sc-146354) and consists of a mixture of three different siRNAs. All three siRNA duplexes target both transcripts. Transient transfections of NIH3T3 cells were performed in 6-Well-Plates (Sarstedt) using ApplifectSI (AppliChem) according to manufacturer’s instructions. In brief, 1.6×10^5^ Cells were seeded per well and transfected using 180 pmol of siRNA as recommended. Cells were harvested after 48 h followed by both RNA and Protein isolation.

## Competing interests

The authors declare that they have no competing interests

## Authors’ contributions

JN designed the experiments and JN, SL, SK and NM performed all the experiments. JN and SL performed the data analysis. JN wrote the manuscript and is responsible for the scientific content of the manuscript. All authors have read and approved the final manuscript.

## Supplementary Material

Additional file 1: Figure S1 Genomic structure of mouse Kbtbd8. **A**: Transcript 1 (T1) consists of 5 exons whereas the start codon is located at the end of the short exon one. T2 consists of exon 3 and 4. The lack of exons 1 and 2 result in a truncated BTB/POZ domain. **B**: RT-PCR analysis on mouse pluripotent cell lines and mouse tissues revealed a ubiquitous expression of both Kbtbd8 transcripts (arrow). HPRT served as loading control. Abbreviations: ESC: Embryonic Stem Cell; maGSC: multipotent adult Germline Stem Cell; iPSC: induced Pluripotent Stem Cell; F9: mouse juvenile teratocarcinoma cell line. Click here for file

Additional file 2: Figure S2 Expression analysis of KBTBD8 on human tissues and cell lines. (**A**) RT-PCR analysis on human cell lines and tissues revealed and ubiquitous expression of KBTBD8. TBP (TATA box binding protein) served as loading control. hFB: human fibroblast cell line BJ. (**B**) BJ cells were fixed and co-stained with KBTBD8 and GM130 or α-Tubulin antibodies. It could be shown that the compartment in which KBTBD8 is localized in human cells is the same as in mouse cells. Scale bars, 10μm. Click here for file
