# Supplementary figures and images for: The novel BTB-kelch protein, KBTBD8, is located in the Golgi apparatus and translocates to the spindle apparatus during mitosis
Source: Cell Div. 2013 Apr 11;8:3. doi: 10.1186/1747-1028-8-3 (PMC3639201; doi:10.1186/1747-1028-8-3)

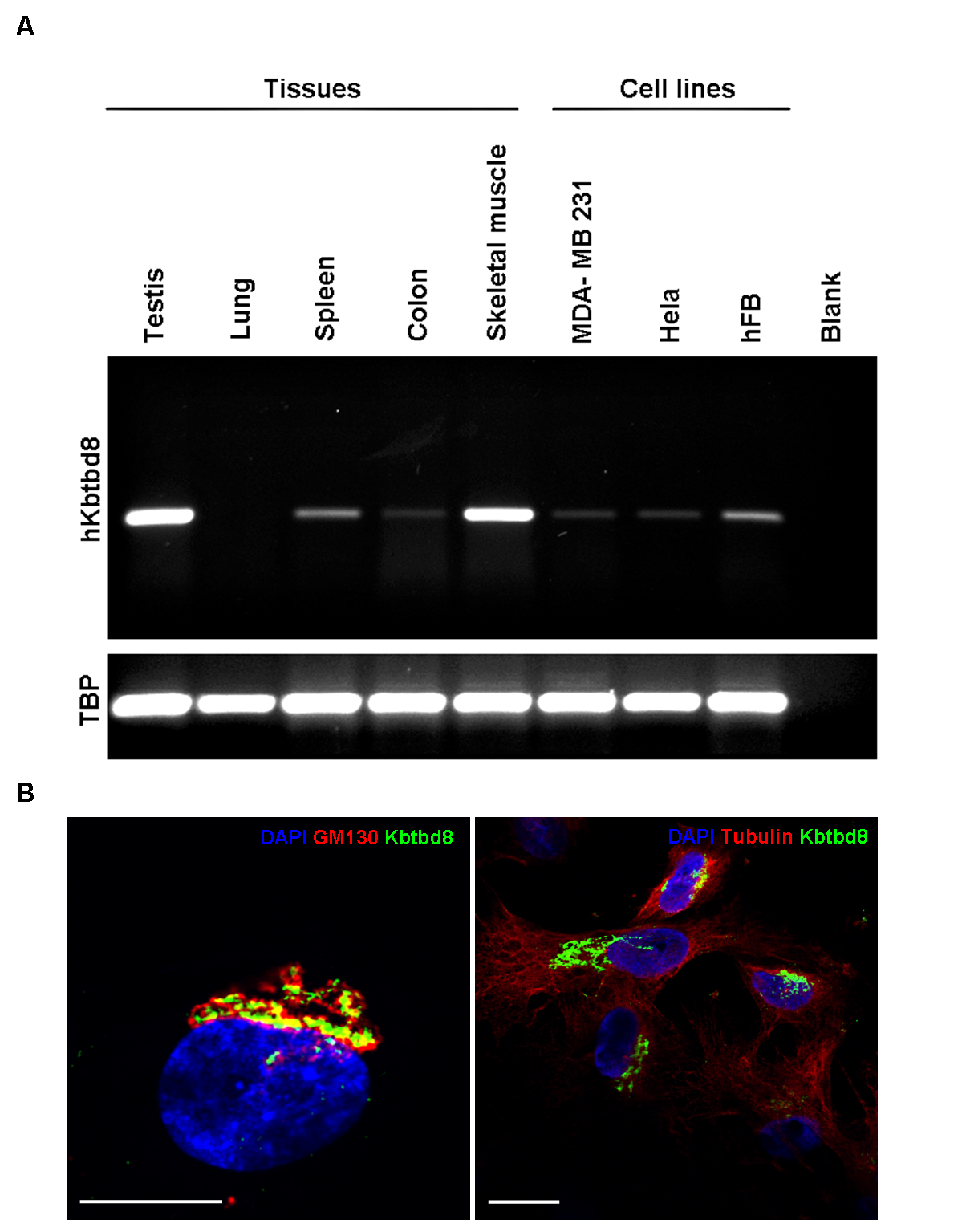

Supplement: Additional file 1: Figure S1 — Genomic structure of mouse Kbtbd8. A: Transcript 1 (T1) consists of 5 exons whereas the start codon is located at the end of the short exon one. T2 consists of exon 3 and 4. The lack of exons 1 and 2 result in a truncated BTB/POZ domain. B: RT-PCR analysis on mouse pluripotent cell lines and mouse tissues revealed a ubiquitous expression of both Kbtbd8 transcripts (arrow). HPRT served as loading control. Abbreviations: ESC: Embryonic Stem Cell; maGSC: multipotent adult Germline Stem Cell; iPSC: induced Pluripotent Stem Cell; F9: mouse juvenile teratocarcinoma cell line. [file 1747-1028-8-3-S1.tiff]

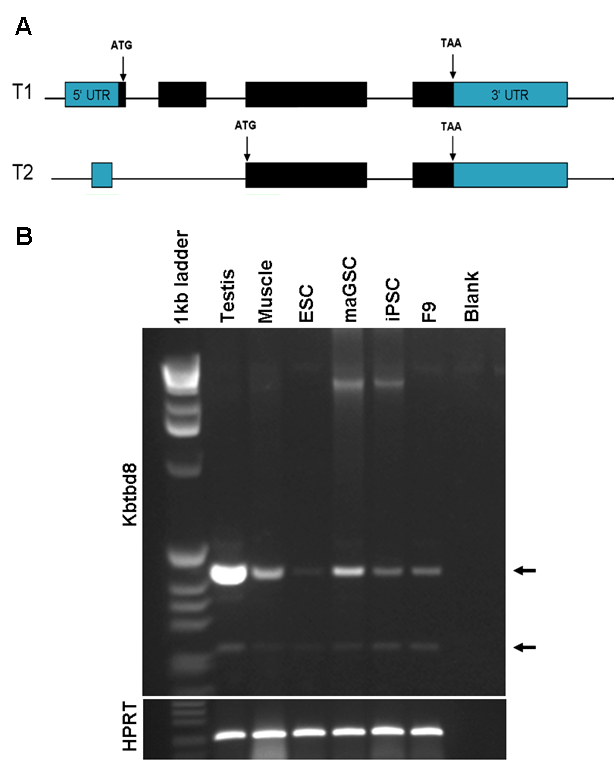

Supplement: Additional file 2: Figure S2 — Expression analysis of KBTBD8 on human tissues and cell lines. (A) RT-PCR analysis on human cell lines and tissues revealed and ubiquitous expression of KBTBD8. TBP (TATA box binding protein) served as loading control. hFB: human fibroblast cell line BJ. (B) BJ cells were fixed and co-stained with KBTBD8 and GM130 or α-Tubulin antibodies. It could be shown that the compartment in which KBTBD8 is localized in human cells is the same as in mouse cells. Scale bars, 10μm. [file 1747-1028-8-3-S2.tiff]
